# Assessment of Adipocyte Transduction Using Different AAV Capsid Variants

**DOI:** 10.3390/ph17091227

**Published:** 2024-09-18

**Authors:** Stanislav Boychenko, Alina Abdullina, Viktor S. Laktyushkin, Andrew Brovin, Alexander D. Egorov

**Affiliations:** 1 Gene Therapy Department, Science Center for Translational Medicine, Sirius University of Science and Technology, 354340 Sirius, Russia; abdullina.ai@learn.siriusuniversity.ru (A.A.); brovin.an@talantiuspeh.ru (A.B.); 2 Resource Center for Cell Technologies, Laboratory Complex, Sirius University of Science and Technology, 354340 Sirius, Russia; laktyushkin.vs@talantiuspeh.ru

**Keywords:** adeno-associated virus, viral tropism, adipocytes, adipose tissue, anti-obesity agents, gene therapy

## Abstract

Background/Objectives: Adeno-associated viruses (AAVs) are widely used as viral vectors for gene delivery in mammalian cells. We focused on the efficacy of the transduction of AAV2/5, 2/6, 2/8 and 2/9 expressing GFP in preadipocyte cells by live imaging microscopy using IncuCyte S3 and flow cytometry. Methods: Three transduction modes in 3T3-L1 preadipocyte cells assessed: AAV transduction in 3T3-L1 preadipocyte cells, transduction with further differentiation into mature adipocyte-like cells and the transduction of differentiated 3T3-L1 adipocytes. For the in vivo study, we injected AAV2/6, AAV2/8 and AAV2/9 in adipose tissue of C57BL6 mice, and the transduction capacity of AAV2/6, along with AAV2/8 and AAV2/9 was evaluated. Results: AAV2/6 demonstrated the highest transduction efficiency in 3T3-L1 preadipocytes, as it was 1.5–2-fold more effective than AAV2/5, and AAV2/8 in the range of viral concentrations from 2 × 10^4^ to 1.6 × 10^5^ VG/cell. AAV2/5 and AAV2/8 showed transduction efficiencies similar to each other. The expression of GFP under the CMV promoter remained stable for up to 20 days. The induction of 3T3-L1 differentiation in three days after AAV transduction did not alter the GFP expression level, and AAV2/6 showed the best transduction efficiency. AAV2/6 demonstrated the ability to transduce mature adipocytes. These results were confirmed by in vivo studies on C57BL6 mice. AAV2/6 had the highest transducing activity on both inguinal and interscapular adipose tissue. Conclusions: Thus, AAV2/6 has demonstrated higher transduction efficacy compared to AAV2/5, AAV2/8 and AAV2/9 both in 3T3-L1 adipocytes and adipose tissue in vivo, which proves its usability along with AAV2/8 and AAV2/9 for gene delivery to adipocytes.

## 1. Introduction

Obesity, which has reached pandemic proportions, is a major contributor to the development of type 2 diabetes mellitus and is a significant risk factor for cardiovascular diseases, including coronary heart disease [1]. The expansion of adipose tissue is characterized by both hypertrophy (increased adipocyte size) and hyperplasia (an increased number of adipocytes), and these processes depend on nutrient influx and the rate of adipocyte differentiation [2]. Adipocyte differentiation, also known as adipo-genesis, is the process by which fibroblast-like precursor cells transform into fully differentiated adipocytes under the influence of adipogenic stimulants such as insulin and glucocorticoid agonists. This process is also influenced by an individual’s genetic background. The action of adipogenic stimuli activates transcription factors that regulate the expression of numerous genes involved in adipocyte differentiation.

Currently, the development of promising therapeutic strategies for obesity is in progress, among which the correction of nutritional habits [3], surgical interventions [4], and the use of most recently approved drugs, such as semaglutide, positively affect the obese and diabetic status [5]. One of the strategies for improving the insulin sensitivity of adipocytes is to influence the molecular mechanisms underlying insulin resistance. The conversion of energy-storing white adipose tissue into energy-burning beige adipose tissue, called browning, has emerged as a promising approach in the fields of metabolic research and obesity treatment [6]. 

As the prominence of the adipose-related research tends to increase, the targeted gene delivery to adipose tissue remains relevant. Adeno-associated viruses (AAVs) are widely used in research and therapeutic applications [7]. Specifically, AAVs are commonly used to introduce transgenes or gene-editing tools into cells and animal models for studying gene function, and disease mechanisms. To date, six AAV vector-based therapies have been approved by European Medicines Agency and Food and Drug Administration, which include Glybera^®^ (uniQure, Amsterdam, The Netherlands) for the treatment of lipoprotein lipase deficiency, Luxturna^®^ (Spark Therapeutics, Inc., Philadelphia, PA, USA) for inherited retinal dystrophy, Zolgensma^®^ (Novartis, Basel, Switzerland) for the treatment of spinal muscular atrophy, Upstaza^®^ (PTC Therapeutics, Inc., Warren Township, NJ, USA) for aromatic L-amino acid decarboxylase deficiency, Roctavian^®^ (BioMarin Pharmaceutical Inc., San Francisco, CA, USA) for hemophilia A, and Hemgenix^®^ (uniQure, Amsterdam, The Netherlands) for hemophilia B [8]. In regard to adipose, such genes were subjected to AAV-mediated delivery as leptin [9,10], BSCL2 [11], BMP7 [12], FGF21 [13], TrkB.FL [14], and evaluated for the treatment of metabolic disorders in vivo.

We decided to perform a comparative analysis of recombinant AAV vectors in order to provide valuable insights into their differential transduction efficiencies and preadipocyte-specific targeting. We chose AAV8 and AAV9 for their known ability to transduce the cells of adipose in vivo [15]. Both for AAV5 [16] and AAV6 [17], the ability to transduce the cells of mesenchymal origin was previously reported.

We propose to use AAVs for gene delivery to adipose tissue cells. Therefore, we decided to utilize a preadipocyte cell model (3T3-L1) in order to evaluate the effects of gene delivery during adipogenesis. The 3T3-L1 cell line is the most investigated model of adipocyte differentiation, widely used for cell-based assays and obesity and insulin resistance-related studies due to its adipogenic capacity [18]. However, it was revealed that AAV transduction capacity for in vitro and in/ex vivo applications differ significantly [19]. Further, it is known that there is a difference between different mammal species in AAV tissue tropism [20]. This is due to the differing distribution of receptors on the membrane and receptor structure. For example, one of the primary receptors that AAV, in particular AAV1, AAV5, and AAV6 serotypes, utilize to transduce cells is sialic acid receptors. In mice, the predominant sialic acid form is N-glycolylneuraminic acid (Neu5Gc). However, humans, due to the lack of the enzyme required to synthesize Neu5Gc, have N-acetylneuraminic acid (Neu5Ac) as the predominant sialic acid. This difference results in the lower efficiency of AAV1, AAV5, and AAV6 in targeting human cells compared to mouse cells [21]. However, in the case of adipocyte transduction by AAV, AAV8 and AAV9 demonstrate strong tropism for adipose tissues in both mouse and human models. Nonetheless, among murine cell lines, 3T3-L1 remains the most used model for the study of metabolic processes in adipose tissue. Here, we show that the transduction capacity of adeno-associated virus serotypes in the 3T3-L1 cell line represents viral tropism of recombinant AAV in vivo.

## 2. Results

### 2.1. Production of Recombinant AAV2/5-GFP, AAV2/6-GFP, AAV2/8-GFP, and AAV2/9-GFP

**Figure 1:**
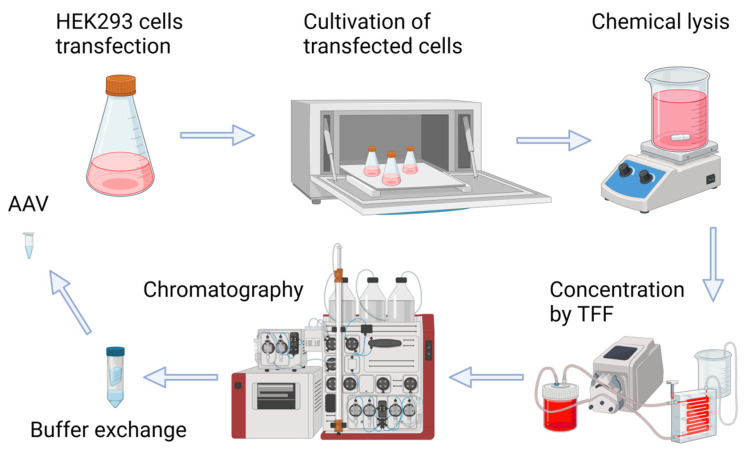
Schematic representation of adeno-associated virus production and purification shows the typical process during AAV manufacturing.

The production of high-quality recombinant viruses and purification procedures constitute an initial step in the study (the process of recombinant AAV production is shown in Figure 1). In brief, the transfection of suspension HEK293 cell culture with three plasmids (pAAV-CMV-GFP, pHelper, and corresponding pRepCap: pRC2/5, pRC2/6, pRC2/8, pRC2/9) resulted in rAAV production. Five days after transfection, the cells were lysed, and the resulting cell lysates containing rAAV particles were subjected to concentration by tangential flow filtration. The concentrated virus was purified by chromatography using CaptureSelect AAVX affinity resin. Further ultrafiltration was performed to exchange buffer and remove excess salt.

The produced rAAV samples were analyzed by means of dynamic light scattering (DLS). The results of the dynamic light scattering analysis (Supplementary Materials) demonstrated the presence of AAV2/5-GFP, AAV2/6-GFP, AAV2/8-GFP, and AAV2/9-GFP particles with hydrodynamic diameters of 26.92 nm, 32.08 nm, 27.49 nm, and 26.62 nm at a concentration of 1.39 × 10^12^, 3.2 × 10^13^, 2.38 × 10^13^, and 4.51 × 10^12^, respectively. The average volume fraction of particles was 99.99%.

The genomic titers of the four rAAV serotypes were determined in the range of 1 × 10^11^–1 × 10^13^ VG/mL (viral genomes per liter of culture), as determined by RT-qPCR. Total viral particle concentrations were 4.6 × 10^11^ (AAV2/5-GFP), 1.64 × 10^11^ (AAV2/6-GFP), 6.98 × 10^12^ (AAV2/8-GFP), and 1.57 × 10^12^ (AAV2/9-GFP) VG/mL.

### 2.2. Fluorescence Intensity in AAV2/6-Transduced 3T3-L1 Cells Is Significantly Higher in Comparison with AAV2/5, 2/8 and 2/9 Serotypes

**Figure 2:**
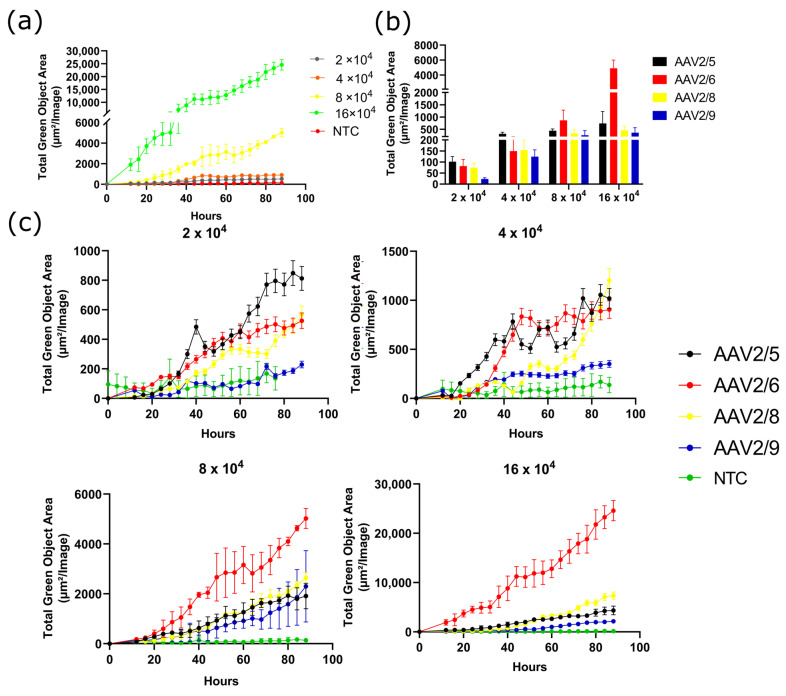
(**a**) Dose-dependent GFP fluorescence intensity measured in Total Green Objects Area (TGOA) during the 4-day time course in 3T3-L1 preadipocyte cells transduced by AAV2/6 at viral concentrations of 2-, 4-, 8-, 16 × 10^4^ VG/cell; (**b**) GFP fluorescence intensity values at 28 h after transduction for the four serotypes; (**c**) A comparison of the transduction efficiencies by the four serotypes (AAV2/5, AAV2/6, AAV2/8, AAV2/9) at viral concentrations of 2-, 4-, 8-, 16 × 10^4^ VG/cell.

The transduction of the 3T3-L1 cell line with AAV2/5, AAV2/6, AAV2/8, and AAV2/9 at four concentrations: 2-, 4-, 8-, and 16 × 10^4^ VG/cell, demonstrated a dose-dependent increase in GFP fluorescence for all serotypes and for AAV2/6 in particular (Figure 2a). AAV2/6 demonstrated much higher GFP fluorescence at all high doses (8 and 16 × 10^4^ VG/cell) at all time points. However, at low doses (2 and 4 × 10^4^ VG/cell), AAV2/6 shows a similar transduction efficiency to AAV2/5 and AAV2/8 (Figure 2b,c). AAV2/8 is second in transduction efficiency only at a concentration of 16 × 10^4^ VG/cell. At lower viral concentrations, AAV2/8 and AAV2/5 show the same transduction activity. Notably, AAV2/9 showed the lowest transduction efficiency among the serotypes tested at all dosages (Figure 2c). Visually distinguishable GFP positive cells on a fluorescent microscope can only be detected at a concentration of AAV2/9 of not less than 8 × 10^4^ VG/cell. Pictures of transduced 3T3-L1 cells taken with the IncuCyte S3 are shown in Figure S1.

### 2.3. Flow Cytometry Demonstrates the Outperformance of AAV2/6-GFP

**Figure 3:**
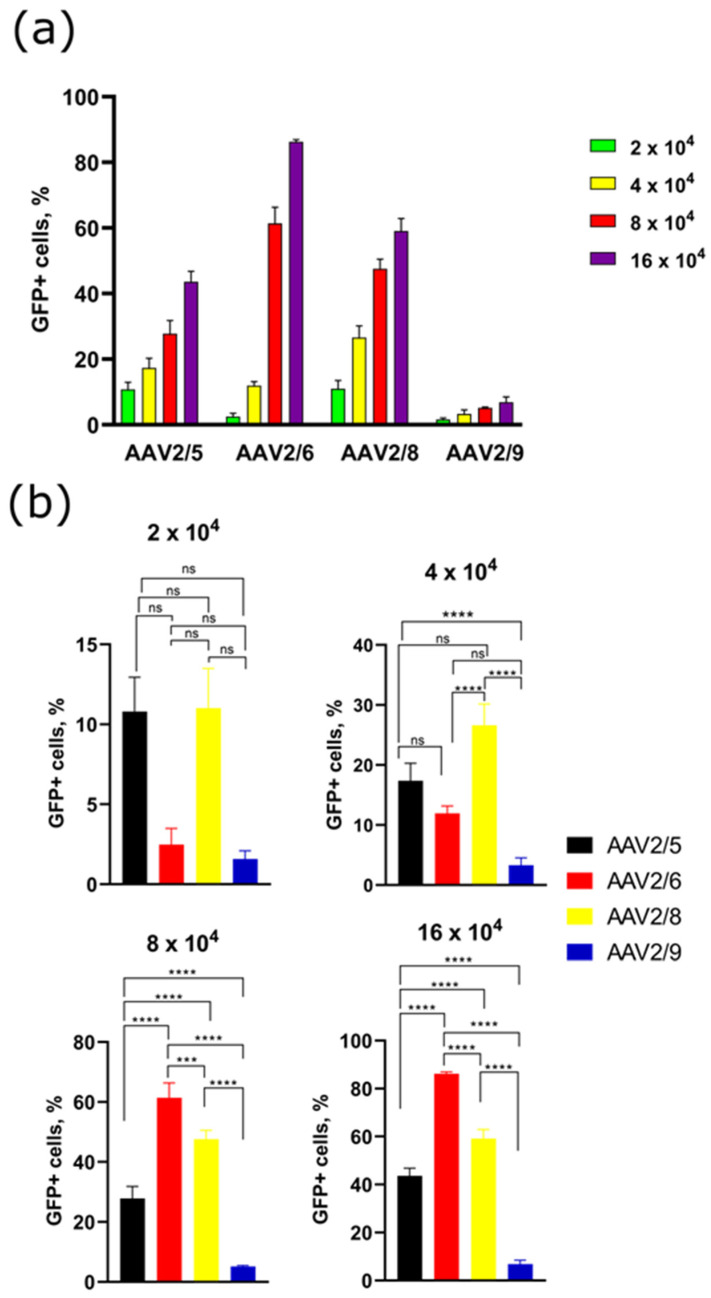
Flow cytometry analysis of 3T3-L1 preadipocyte cells transduced by AAV2/5, AAV2/6, AAV2/8, and AAV2/9 (four days after transduction). (**a**) Dose-dependent percentage of GFP-positive 3T3-L1 cells transduced by AAV2/5, AAV2/6, AAV2/8, and AAV2/9 at four viral concentrations: 2 × 10^4^ (green bar), 4 × 10^4^ (yellow bar), 8 × 10^4^ (red bar), 8 × 10^4^ (purple bar); (**b**) statistical analysis of GFP+ 3T3-L1 cells at four viral concentrations. (***) *p*-value < 0.001, (****) *p*-value < 0.0001, not significant (ns) *p* > 0.05.

Four days after transduction, the number of GFP-positive cells was assessed by flow cytometry (Figure 3). Untreated 3T3-L1 cells were used as a negative control (see Figure S2, which presents the strategy of gating). After transduction with AAVs at the lowest concentrations (2 × 10^4^ VG/cell), the differences observed in fluorescence intensity between cells transduced with different serotypes were statistically not significant, which is consistent with the GFP fluorescence intensity detected with Incucyte. We noticed that, as a result of AAV2/6 transduction, the fluorescence intensity changed in a different manner compared to other evaluated AAV serotypes. In particular, the transduction efficiency of AAV2/6-GFP arose drastically at concentrations higher than 4 × 10^4^ VG/cell in contrast to the rest of the samples (Figure 3b). At a concentration of 4 × 10^4^ VG/cell, a significant difference in the number of GFP+ cells was observed between AAV2/6 and AAV2/8 (*p*-value < 0.0001), AAV2/5 and AAV2/9 (*p*-value < 0.0001), AAV2/8, and AAV2/9 (*p*-value < 0.0001). Moreover, AAV2/9 had the lowest transduction efficiency among the AAV serotypes tested. At 8- and 16 × 10^4^ VG/cell, AAV2/6 showed better performance, with a significant difference (*p*-value < 0.0001) compared to other serotypes. AAV2/8 and AAV2/5 are similar to AAV2/6 in terms of transduction activity (Figure 3b).

### 2.4. GFP Fluorescence Intensity Peaks 15 Days after Transduction for All Four Serotypes

**Figure 4:**
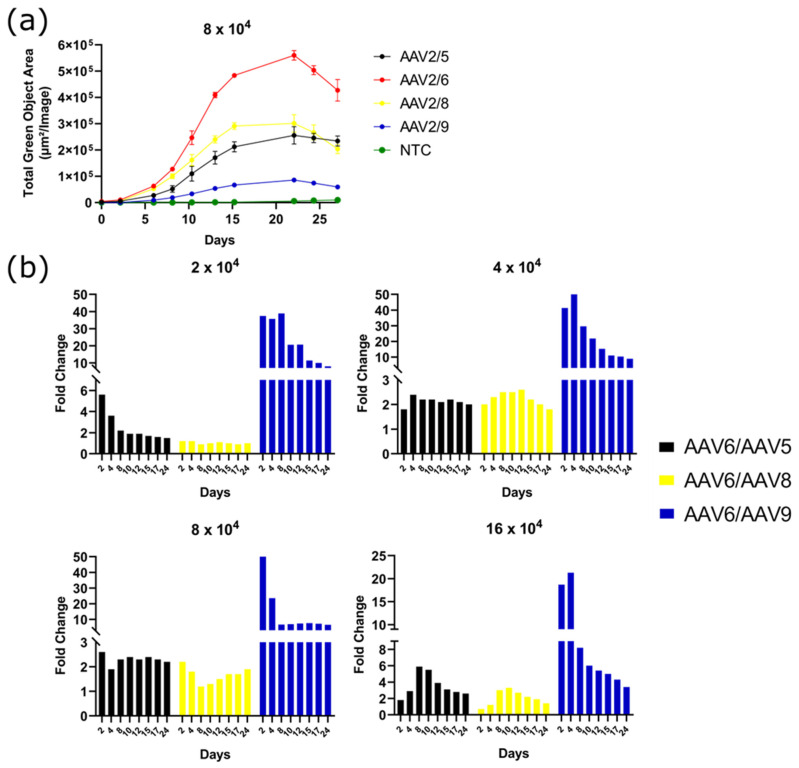
(**a**) GFP fluorescence intensity measured in Total Green Objects Area (TGOA) during the 25-day time course in 3T3-L1 preadipocyte cell transduced by AAV2/5, AAV2/6, AAV2/8, and AAV2/9 at viral concentration of 8 × 10^4^ VG/cell; (**b**) GFP fluorescence intensity fold change differences between AAV2/6 and AAV2/5, AAV2/8, or AAV2/9 at a viral concentrations of 2-, 4-, 8-, and 16 × 10^4^ VG/cell.

To determine the period during which different AAV serotypes were able to maintain transgene expression, GFP fluorescence intensity was measured for a month after transduction. The fluorescence intensity reached its maximum 15 to 20 days after transduction. The pattern of fluorescence change was similar for all the AAV serotypes tested. After 25 days, there was a notable decrease in GFP fluorescence intensity, which could have possibly happened both due to cell death and due to GFP bleaching (Figure 4a). The level of GFP fluorescence as a result of AAV2/6 transduction of 3T3-L1 cells throughout the course of analysis at four viral concentrations was 2–6 times higher than that of serotype AAV2/5, 2–4 times higher than that of serotype AAV2/8, and 4–50 times higher than that of AAV2/9 (Figure 4b). Moreover, the difference in GFP fluorescence intensity was maximal in the period of 4 days after transduction and further decreased or insignificantly changed.

### 2.5. Induction of 3T3-L1 Adipogenic Differentiation after Transduction

**Figure 5:**
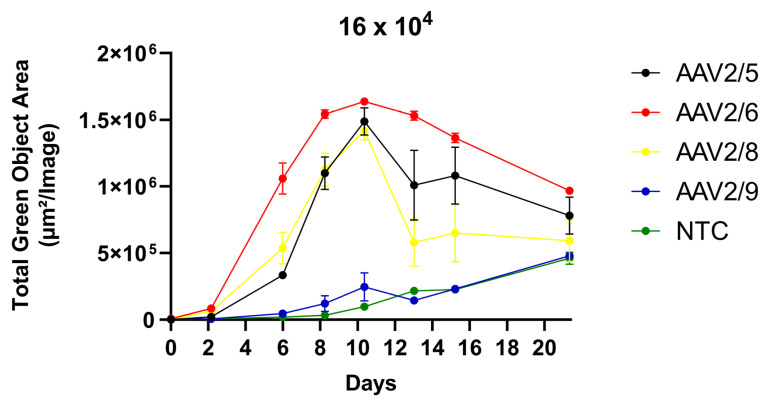
GFP fluorescence intensity measured in Total Green Objects Area (TGOA) during the 25-day time course in 3T3-L1 preadipocyte cell transduced by AAV2/5, AAV2/6, AAV2/8, AAV2/9 at viral concentration of 16 × 10^4^ VG/cell. Induction of 3T3-L1 adipogenic differentiation was made at one day post-transduction.

The induction of 3T3-L1 differentiation in adipocytes alters their phenotype, i.e., not only morphology but the presence of receptors on the surface. Thus, the differentiation can change AAV tropism, and subsequently transgene expression levels. To assess this effect, we induced differentiation with IBMX-DEX-INS solution one day after AAV transduction. AAV2/6 has the highest transducing activity (Figure 5). GFP fluorescence intensity peaks 10 days after transduction, which is 5 to 10 days less in the case of no differentiation. Pictures of transduced 3T3-L1 cells taken with the IncuCyte S3 are shown in Figure S3.

### 2.6. AAV2/6 Serotype Has Higher Transduction Capacity in Differentiated 3T3-L1 Cells Compared with AAV2/5, AAV2/8 and AAV2/9

**Figure 6:**
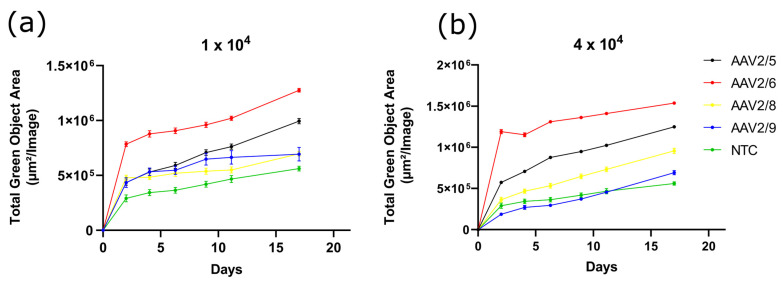
GFP fluorescence intensity measured in Total Green Objects Area (TGOA) during the 17-day time course in differentiated 3T3-L1 cells transduced by AAV2/5, AAV2/6, AAV2/8, AAV2/9 at viral concentrations of 1 × 10^4^ (**a**) and 4 × 10^4^ (**b**) VG/cell.

To study the ability of AAV to transduce differentiated preadipocytes, 3T3-L1 cells were differentiated with an IBMX-DEX-INS cocktail before transduction. Cells were transduced with AAV in 14 days after the induction of differentiation. The AAV transduction efficiency of differentiated 3T3-L1 cells was found to be significantly lower than that of preadipocytes. AAV2/9 is practically unable to transduce 3T3-L1 adipocyte-like cells (Figure 6). Pictures of transduced 3T3-L1 cells taken with the IncuCyte S3 are shown in Figure S4. Nile-Red-stained differentiated 3T3-L1 cells are shown in Figure S5.

### 2.7. AAV2/6 Serotype Shows a Better Adipose Tissue Transduction Ability In Vivo

**Figure 7:**
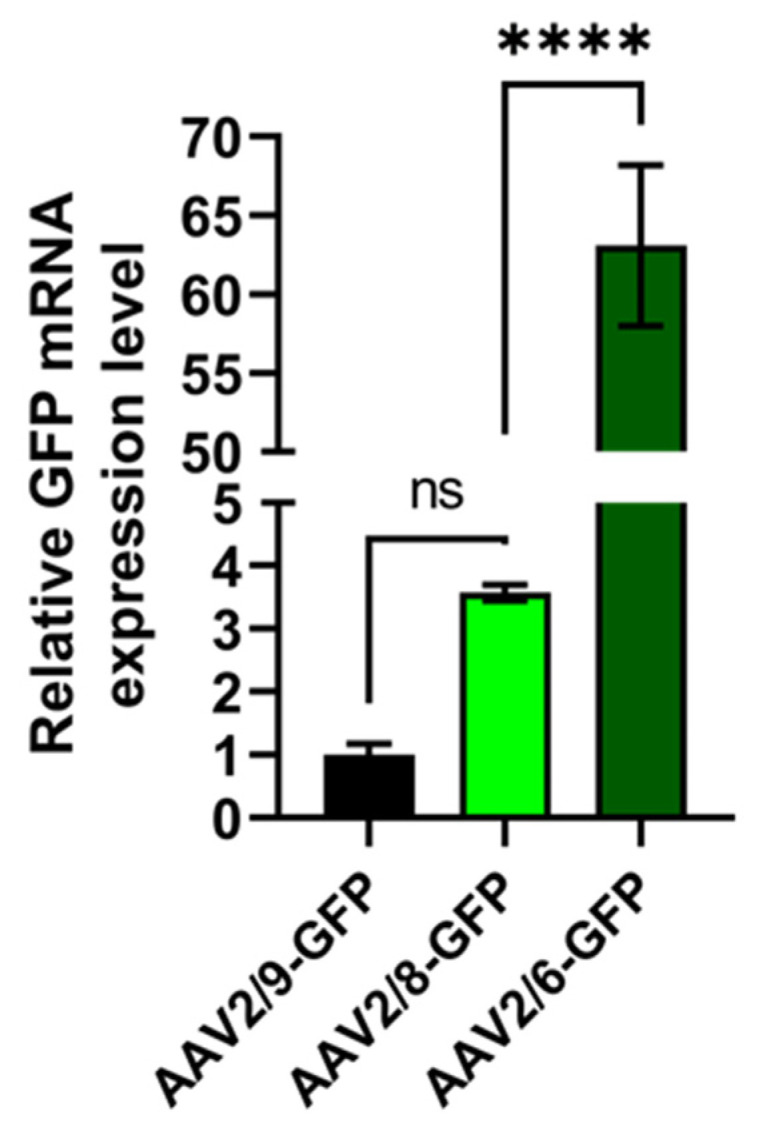
RT-qPCR analysis of GFP expression in the adipose tissue samples from C57BL6 mice. Gene of interest—GFP; Ct values are normalized by the endogenous control gene—Gapdh; (****) *p* < 0.0001, not significant (ns) *p* > 0.05.

The evaluation of AAV tropism in vivo was performed using several known AAVs of different serotypes (2/6, 2/8, 2/9) coding for GFP on C57BL6 mice. In order to estimate the transduction efficiency, 100 μL of each recombinant AAV product was injected in adipose tissue of the inguinal region. One week after injection, adipose tissue was subjected to postmortal manipulations: samples were extracted for both confocal microcopy and RNA analysis. For PCR analysis, 50 mg of adipose was used for RNA extraction. The results of the PCR analysis are presented at Figure 7. Samples of adipose tissue extracted from AAV2/9-treated mice were used as a reference due to them having the lowest Ct value. We see the four-fold increase in relative expression of GFP in adipose of AAV2/8-injected mice, which had no statistical significance. Instead, in sample AAV2/6-injected mice, there was 65-fold increase in the relative expression of GFP compared to AAV2/9, and a 16-fold increase in AAV2/8, which was statistically significant. These results strictly confirm the data we earlier obtained in vitro in the 3T3-L1 cell line.

**Figure 8:**
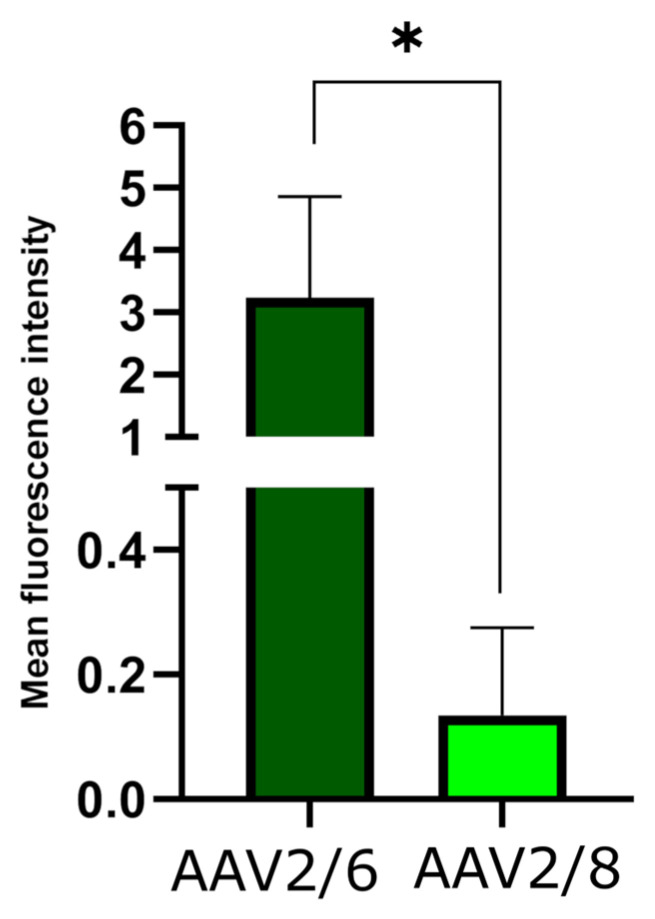
Mean fluorescence intensity of Katushka2S in the interscapular adipose tissue. (*) *p*-value < 0.05.

To assess the transduction capacity of AAV2/6 and AAV2/8 on brown adipose tissue in vivo, 100 μL (5 × 10^11^ VG) of recombinant AAV2/6 and AAV2/8 coding for the fluorescent protein Katushka2S under brown adipose-specific *Ucp1* promoter were injected into the interscapular region of C57BL6 mice. Interscapular adipose was extracted one week after the administration of viral vectors. Katushka2S fluorescence intensities were analyzed using confocal microscopy (Figure 8). Confocal fluorescence images of the interscapular adipose tissue are shown in Figure S5. Mean fluorescence intensity of AAV2/6-injected adipose samples was more than 10 times higher in comparison with AAV2/8-injected adipose, which indicates the higher tropism of AAV2/6 to brown adipose tissue.

## 3. Discussion

Virus capsids are substantial for AAV-mediated gene transfer, since they determine the host–cell tropism, immunogenicity, and transduction efficiency. Different AAV serotypes have been identified by the transduction efficiency attributed to the capsid [22]. As capsid is a primary interface between the host and the vector, its features play a crucial role in various stages of the gene transfer process: binding to receptors, internalization, and trafficking within the cell [23].

Among the natural serotypes isolated, many of which exhibit unique patterns of tissue tropism, the transduction capabilities of several vectors were noticed. The comparative analysis of recombinant AAV vectors has provided valuable insights into their differential transduction efficiencies and tissue-specific targeting.

Intriguingly, several studies highlighted the superiority of the AAV6 serotype for the cells of mesodermal origin. The identification of the receptors on the target cells that mediate cellular tropism effects [24] demonstrated that the high transduction efficacy of AAV6 for the skeletal and cardiac tissue compared to AAV8 and AAV9 is due to the involvement of the α2,3- or α2,6-linked sialic acid receptor for the cellular entry. The AAV6 uses the α2,3- or α2,6-linked sialic acid receptor, whereas AAV8 and AAV9 use laminin receptors. It was also discovered that α2–6sialylation is present on a surface of adipose-derived mesenchymal stem cells, and may serve as a marker of their differentiation potential [25].

We use AAVs for the transduction of adipose tissue cells, and we aimed to choose a relevant cell model to evaluate the effects of gene therapy on adipogenesis. Therefore, we focused on the efficacy of transduction of AAV2/5, AAV2/6, AAV2/8 and AAV2/9 in the widely utilized murine preadipocyte cell line 3T3-L1. Here, we evaluated the efficacy of transduction of AAV 2/5, 2/6, 2/8, 2/9 expressing GFP in 3T3-L1 murine preadipocyte cells by live imaging microscopy using IncuCyte S3 and flow cytometry. Since all the AAV vectors used in this study share the same AAV2 ITR, then observed differences in the transduction efficiency of AAV serotypes for 3T3-L1 murine preadipocyte cells can be attributed to the difference in capsid proteins.

Three transduction modes were assessed: AAV transduction in 3T3-L1 preadipocyte cells with or without further differentiation into mature adipocytes and transduction in differentiated 3T3-L1 adipocyte cells. The differentiation of 3T3-L1 was induced by adipogenic IBMX-DEX-INS cocktail. AAV2/6 demonstrated the superior transduction efficiency in 3T3-L1 preadipocytes in the range of viral concentration from 2 × 10^4^ to 16 × 10^4^ VG/cell (Figure 1). AAV2/6 demonstrated the highest transduction efficiency in 3T3-L1 preadipocytes, as it was 1.5–2-fold more effective than AAV2/5 and AAV2/8 in the range of viral concentration from 2 × 10^4^ to 8 × 10^4^ VG/cell. AAV2/8 showed a better transduction efficiency than AAV2/5 at high dosage (8 and 16 × 10^4^ VG/cell) and a similar efficiency at lower dosage. The different levels of GFP fluorescence at low and high doses between serotypes AAV2/5, AAV2/6, and AAV2/8 can be explained by the difference in receptors that mediate entry into host cells. For AAV5, the primary receptor is N-linked sialic acids, and the co-receptor is platelet-derived growth factor receptor (PDGFR) [26]. For AAV6, in addition to the N-linked sialic acid, heparan sulfate and proteoglycans serve as primary receptors, and the list of co-receptors includes GPR108 and EGFR. The presence of the primary and co-receptors at the surface determines the level of saturation for different AAV serotypes, for each is unique. Theoretically, after saturation, there will be no further increase in transduction efficacy.

Despite the fact that, previously, AAV8 [26] was used for gene delivery in the 3T3-L1 preadipocyte cell line, our study shows that AAV2/6 is effective in 3T3-L1 preadipocyte cells. We showed that the transduction efficiency of AAV2/6 was pronounced in comparison with AAV2/5, AAV2/8 and AAV2/9. AAV2/5 and AAV2/8 showed a lower transduction efficiency, with values similar to each other.

Ensuring prolonged transgene expression is one of the main objectives of gene therapy. Thus, the aim of our study was also to evaluate the duration of transgene expression in 3T3-L1 preadipocytes and adipocytes after differentiation. We demonstrated that the expression of GFP under the control of CMV promoter remained stable for up to 20 days in preadipocytes for all tested AAV serotypes. In differentiated 3T3-L1 cells, GFP fluorescence reached a plateau 15 days after transduction. The induction of 3T3-L1 differentiation in three days after AAV transduction did not alter much the GFP expression level, and AAV2/6 showed the highest transduction efficiency. However, without differentiation, the GFP fluorescence level reaches a maximum on day 20 after transduction, whereas after the induction of differentiation, a maximum was reached on day 10, followed by a decline in fluorescence level. In this way, adipogenesis affects the duration of AAV-mediated transgene expression.

To confirm the tissue tropism of AAV2/6, we performed a series of in vivo studies on C57BL6 mice. We observed a greater transduction ability of AAV2/6, which was confirmed by quantitative PCR and confocal microscopy for inguinal adipose tissue, and by confocal microscopy for interscapular adipose.

AAV2/6 demonstrated the ability to transduce mature adipocytes. Thus, AAV2/6, compared to AAV2/5 and AAV2/8, demonstrated higher transduction efficacy in 3T3-L1 preadipocytes and mature adipocytes, which proved its usability along with AAV2/8 and AAV2/9 for gene delivery to adipocytes.

## 4. Materials and Methods

### 4.1. HEK293 Suspension Culture

Suspension cell cultures were grown on a BalanCD HEK293 animal component-free chemically defined medium (Irvine Scientific, Santa Ana, CA, USA) in a cell concentration range between 1 × 10^5^ and 3 × 10^6^ cells/mL. HEK293 cells were cultured in plastic Erlenmeyer flasks (Corning, Corning, NY, USA) inside the INFORS HT Multitron orbital shaker–incubator (INFORS, Bottmingen, Switzerland) at +37 °C, 5% CO2, 80% humidity, 100 rpm. Cells were passaged twice a week by the addition of fresh medium to reach 1 × 10^5^ cells/mL. Alternatively, suspension cell cultures were passaged by centrifugation with subsequent resuspension in order to remove waste products.

### 4.2. Adeno-Associated Virus Production

Adeno-associated viral vectors (AAVs) were produced by the transfection of suspension HEK293 cells. Suspension cell cultures were grown up to concentrations of 1 × 10^6^ cells/mL. Transfection was performed using PEI (Linear, MW 40,000, Polysciences Inc., Warrington, PA, USA) and the following DNA plasmid vectors (pDNA): pAAV-CMV-GFP and pAAV-UCP1-Katushka2S coding for Katushka2S, pHelper, and the corresponding pRC vector. After the sequential addition of plasmids into the centrifuge tube, the solution of PEI 1 μg/mL was prepared in a mass ratio of pDNA:PEI 1:5. After this preparation, components were diluted by a serum-free medium (either Opti-MEM or DMEM, Invitrogen, Carlsbad, CA, USA), and the contents of the tubes were mixed by pipetting and kept at room temperature for 10 min. For transfection, a prepared mixture with a concentration of plasmids of 1.5 μg per million cells was added to the Erlenmeyer flasks. Previously prepared transfection mixtures were added directly into the flasks using a single-channel dispenser. After this, the flasks were transferred to an incubator for cultivation and kept at 37.0 °C, with a humidity of 70%, a CO2 content of 5%, and stirring at 120 rpm. RepCap vectors (pRC) contained the same Rep but different Cap sequences, coding for AAV2/5 (pAAV-RC2/5), AAV2/6 (pAAV-RC2/6), AAV2/8 (pAAV-RC2/8), and AAV2/9 (pAAV-RC2/9) capsid proteins for the packaging of different recombinant AAV serotypes.

After 120 h, post transfection chemical lysis of the cell suspension was carried out using 10% Tween 20 for 1 h at 37 °C and stirring 130 rpm. After chemical lysis, enzymatic treatment with Serratia marcescens endonuclease was performed in order to hydrolyze free nucleic acids. The resulting lysates were clarified by adding diatomaceous earth (0.01 g/mL of Celite HyFlo Super Cel, Roth), mixing for 5 min at 37 °C and 330 rpm, followed by the sterilizing filtration of the lysates through a filter with a pore diameter of 0.22 μm. The tangential filtration of the clarified lysates of AAV2/5-, AAV2/6-, AAV2/8- and AAV2/9-GFP was performed using VivaFlow200 tangential filtration system (Sartorius, Bohemia, NY, USA). Chromatographic purification was carried out using the CaptureSelect POROS AAVX affinity resin (ThermoFischer Scientific, Waltham, MA, USA) with Bio-Rad Quest 10 Plus system. Finally, recombinant AAV vectors were concentrated to a volume of 1 mL through dialysis in a 1x phosphate-buffered saline (PBS) (0.37 M NaCl) using Vivaspin 20 ultrafiltration units (Sartorius, Bohemia, NY, USA), and 0.001% Pluronic F-68 was added (Sigma-Aldrich, Gillingham, UK).

### 4.3. Determination of Titer for AAV Genomes (RT-qPCR)

The genomic titers of rAAV serotypes were determined by quantitative PCR (qPCR). The Biomaster HS-qPCR-Hi-ROX mix (Biolabmix LLC, MHR020-2040, Novosibirsk, Russia) contained ITR sequence-specific forward (5′-*GGAACCCCTAGTGATGGAGTT*-3′) and reverse (5′-*CGGCCTCAGTGAGCGA*-3′) primers and a fluorescent probe (5′-FAM-*CACTCCCTCTCTGCGCGCTCG*-BHQ1-3′). The components were mixed at the following ratio: 7.5 μL of Biomaster HS-qPCR-Hi-ROX mix, 1 μL H_2_O, 50 μM of each oligonucleotide, and 5 μL of DNA. Prior to analysis, the samples containing rAAV were treated with DNase I (Biolabmix LLC, EM-250) according to the manufacturer’s instructions, mixing 2 μL of the sample with two units of DNase I and a one-time buffer (100 mM Tris-HCl (pH 7.0), 30 mM MgCl_2_, 30 mM CaCl_2_) in a final volume of 20 μL, followed by a 30 min incubation at +37 °C and enzyme inactivation for 15 min at +55 °C to remove non-viral DNA. Next, 20 μL of Proteinase K diluted to a concentration of 0.1 U/μL was added to the sample and incubated for 1 h at +55 °C following enzyme inactivation at +95 °C for 5 min. The resulting DNA preparation was used for qPCR analysis using a standard curve. A linearized pAAV-GFP plasmid was used to construct the standard curve. A series of serial dilutions were carried out, starting with 1.8 ng/μL (corresponding to 0.2 × 10^8^ gene copies/μL) in 10-fold increments down to 0.02 fg/μL (corresponding to 0.2 × 10^4^ gene copies/μL), the lower limit of detectable concentrations. PCR analysis was carried out using a StepOnePlus device (Thermo Scientific, Waltham, MA, USA) using the Quantification-Standard Curve application of the StepOneSoftWare V2.3 software. The standard curve was considered reliable if the coefficient of determination (R2) exceeded 0.99 and the reaction efficiency was in the range of 85 to 100%. Reliable concentration measurements were made in the range from the seventh to the twenty-fifth cycle, which corresponds to intermediate and boundary points of the standard curve in the range of determined concentrations.

### 4.4. Sample Analysis Using Dynamic Light Scattering (DLS)

Dynamic light scattering (DLS) is a non-contact method that applies the light scattering effect and is designed to measure the size of the nano- and submicron particles of a dispersed phase that have Brownian motion. The DLS method possesses an advantage over other optical methods by allowing the sample to be measured in its native form. The samples were measured at +25 °C on a Zetasizer Ultra (Malvern Panalytical Ltd., Malvern, UK) analyzer equipped with a He-Ne laser with a wavelength of 633 nm and a maximum power of 10 mW. The multi-angle light scattering method, based on the sequential capture of the analytical signal from three detection angles of scattered radiation, allowed for the estimation of the hydrodynamic diameter, the modality of particle distribution, and the fractional ratios. The small-volume quartz cuvette (Malvern Panalytical Ltd., Malvern, UK) was used for measurements. The data were processed using ZS XPLORER software v3.1.0 (Malvern Panalytical Ltd., Malvern, UK).

### 4.5. 3T3-L1 Cell Culture

Cells were grown on Dulbecco’s modification of Eagle’s medium (DMEM high glucose, 4.5 g/L, Paneco, Moscow, Russia) with an addition of 10% bovine calf serum (NBCS New Zealand origin, Gibco, Thermo Fisher Scientific, Waltham, MA, USA) and 2 mM L-glutamine (Paneco, Moscow, Russia). 3T3-L1 cells were seeded onto cell-culture-treated plastic dishes, and incubated at 37 °C in a humid modified atmosphere (95% air, 5% CO_2_). Cells were passaged once a week until reaching approximately 70% confluence, and the medium (DMEM+10%BCS) was replaced every 3 days. For 3T3-L1, is important to avoid cell-to-cell contact unless required by the experimental conditions. Pictures of undifferentiated 3T3-L1 cells and Nile-Red-stained differentiated 3T3-L1 cells are shown in Figure S6.

### 4.6. AAV Transduction

After seeding the cells into a 48-well culture plate for flow cytometry analysis and a 24-well for analysis in a IncuCyte S3, the volume of viral eluate required was calculated (based on the cell number, in a range from 10,000 to 300,000 viral genomes per cell). Thus, to transduce 100,000 cells with 40,000 viral genomes per cell, 4 × 10^9^ viral genomes were introduced into a well. The required volume of viral eluate was added immediately after seeding unless required by the experimental conditions. The 3T3-L1 cell line was transduced in DMEM high glucose with 10% NBCS.

### 4.7. Induction of Adipogenic Differentiation

Cells were cultured up to 100% confluence in the DMEM+10% NBCS medium, and differentiation was induced 48 h after reaching confluency. To induce differentiation, the medium was replaced by DMEM medium with 10% fetal bovine serum (FBS South American origin, Gibco, Thermo Fisher Scientific, Waltham, MA, USA) and with the addition of 0.5 mM isobutymethylxanthine (Merck KGaA, Darmstadt, Germany), 1 μM dexamethasone (Merck KGaA, Darmstadt, Germany), 5 μg/mL insulin (Paneco, Moscow, Russia), mentioned as IBMX-DEX-INS. After 48 h of induction, the cells were cultured in DMEM with 10% FBS and 1 μg/mL insulin (adipocyte maintenance medium); the medium was replaced every 2 days.

### 4.8. Flow Cytometry

Ninety-six hours post-transduction, the number of GFP-positive cells was analyzed by flow cytometry. Briefly, the cells were trypsinized, washed twice with 500 μL PBS, and resuspended in 250 μL chilled FACS buffer (1 × PBS, 2% FBS, 1 mM EDTA). The data were recorded on the CytoFLEX B2-R2-V0 flow cytometer (Indianapolis, IN, USA) using CytExpert software v1.2 gating on single FITC-positive live cells. The analysis of median fluorescent intensity (MFI) values of GFP-positive populations was carried out using FlowJo™ v10 software.

### 4.9. RNA Extraction and Reverse Transcription

RNA extraction was carried out with Lira+ kit (LRP-100-3, Biolabmix, Novosibirsk, Russia) and reverse transcription was carried out with RT-M-MuLV-RH kit (R01-250, Biolabmix, Novosibirsk, Russia). An amount of 1 μg of total RNA and 3 μL random hexa primer, diluted in deionized nuclease free water up to 18 μL, was used for primer annealing. In the first stage, the mixture was heated at 70 °C for 3 min and thawed on ice for the melting of the RNA second structures and primer annealing. In the second stage, 12 μL of reservation mix (6 μL of 5хKCl buffer, 3 μL of DTT, 1.5 μL 20× dNTP, 1.5 M-MuLV revertase) was added to RNA prepared samples. The final mixture was incubated for 10 min at 25° C, 60 min at 42 °C, and 10 min at 70 °C in T100 Thermal Cycler (1861096, BioRad, Hercules, CA, USA).

### 4.10. Gene Expression Analysis

For gene expression, RT-PCR analysis was used, with the following components: HS-qPCR SYBR Blue kit (MHC030-2040, Biolabmix, Novosibirsk, Russia), primers for PPIA (forward: CATTATGGCGTGTAAAGTCAC, reverse: CAGACAAAGTTCCAAAGACAG) GFP (forward: AAGCTGACCCTGAAGTTCATC, reverse: CAGGACCATGTGATCGCG) and prepared cDNA samples. cDNA concertation was normalized by similar values of the PPIA threshold cycles. The PCR mix was carried out with 10 ng of cDNA per reaction, 10 μL of 2x HS-qPCR SYBR Blue premix, 0.25 pmole of primers per one reaction and deionized nuclease free water added up to 20 μL. The 2-step PCR reaction was performed with the following stages: 5 min preheating, 40 cycles of 10 s denaturation and 30 s annealing, combined with elongation and a final melt-curve stage analysis of the reaction products. RT-PCR analysis was conducted with the StepOnePlus real-time PCR system (437659, Thermo Scientific, USA) and the 2ddCt relative expression measurement mode in the StepOneSoftWare V2.3 software. One-way statistic ANOVA analysis was performed with GraphPad Prism 9.3.1.

### 4.11. Live Imaging Microscopy IncuCyte S3

Live imaging was performed using the IncuCyte S3 system (Sartorius AG, Göttingen, Germany) in the bright field and GFP channels (300 ms exposure time), taking images every 2 h for 4 days. The intensity of GFP expression was presented as Total Green Objects Area (μm^2^/image) (TGOA) values. In the longitudinal experiments, differentiation images were taken every 2 days (48 h).

After transduction, the culture plate was transferred to the IncuCyte S3 system for intravital cell analysis. The system selected the scan type according to schedule (scan on schedule). In the Create or Restore Vessel field, the corresponding type of vessel used (in our case, a 48-well plate manufactured by Eppendorf) and imaging channels (Brightfield and Green) were input. In the Vessel Location field, the position in the system corresponding to the position of the scanned tablet was selected. In the Scan Pattern field, the wells of the plate to be analyzed and the number of microphotographs for each well of the plate (9) were selected. In the Vessel Notebook field, the name of the experiment in the system, as well as the cell type, was entered. In the Analysis Setup field, the following settings are selected: Basic Analyzer, GFP per cell. In the Scan Schedule field, a scanning schedule was generated for the analyzed wells of the plate.

### 4.12. Confocal Microscopy

Confocal fluorescence images were obtained using an inverted point scanning confocal microscope (LSM 980 Airyscan based on Axio Observer 7, Carl Zeiss Microscopy GmbH, Jena, Germany) equipped with a motorized piezo stage through a 10× objective lens (EC Plan-Neofluar, numerical aperture 0.3, Carl Zeiss Microscopy GmbH, Jena, Germany). Images were obtained in Airyscan mode. The GFP was excited by a 488 nm laser diode (maximum power: 13 mW; AOTF transmission was set to 0.9%). Emission light was passed through the emission filter of the Airyscan (495–550 nm). Detector gain was set to 850 V. Confocal scan zoom was set to 2×, and image size was set to 1045 × 1045 pixels. The image parameters were set as follows: pixel dwell time, 1.96 micro s (scan speed 6); pixel size, dx = dy = 0.39 µm. Raw Airyscan images were processed by the Airyscan processing algorithm (processing strength 3.9). Images were obtained using Zen software (version Zen Blue 3.2, Carl Zeiss Microscopy GmbH, Jena, Germany).

### 4.13. Statistical Analysis

Statistical analysis of the normal distribution of samples and the confidence interval of differences for rejection of the null hypothesis was performed using GraphPad Prism 8.2.1 software (Shapiro–Wilk test, ordinary one-way ANOVA, Sidak’s multiple comparison test). Results are presented as mean ± standard deviation of 2–3 biological replicates; for the confidence interval, the levels of difference are listed as follows: significant (**)–*p*-value < 0.01, (***) *p*-value < 0.001, (****) *p*-value < 0.0001, not significant (ns)–*p* > 0.05.

## 5. Conclusions

AAV2/6, compared to AAV2/5, AAV2/8 and AAV2/9, demonstrated the higher transduction efficacy both in vitro in 3T3-L1 preadipocytes and mature adipocytes. For the in vivo study in adipose tissue of C57BL6 mice, we injected AAV2/6, AAV2/8 and AAV2/9 in C57BL6 mice, and the evaluation has proved the higher transduction capacity of AAV2/6 and its usability, along with AAV2/8 and AAV2/9 for gene delivery to adipose tissue. Taking into account the differences in the tissue tropism of different AAV serotypes, we recommend the use of AAV2/6 for in vitro and in vivo applications, as well as targeted gene delivery to both preadipocytes and adipocytes.

## Data Availability

The original contributions presented in the study are included in the article/Supplementary Material, further inquiries can be directed to the corresponding author.

## Supplementary Materials

The following supporting information can be downloaded at: https://www.mdpi.com/article/10.3390/ph17091227/s1, Figure S1. 3T3-L1 cells transduced cells with different AAV serotypes. AAV concentration −16 × 10^4^ MOI. Images of 3T3-L1 cells taken with the IncuCyte S3 in 48 h after transduction. Figure S2. Gating of cell populations expressing GFP. Untreated 3T3-L1 cells (NTC) were used as a negative control. Figure S3. 3T3-L1 cells transduced cells with different AAV serotypes with further induction of differentiation. AAV concentration −16 × 10^4^ MOI, 10 days after transduction. Images of 3T3-L1 cells taken with the IncuCyte S3. Figure S4. Differentiated 3T3-L1 cells transduced cells with different AAV serotypes. AAV concentration −4 × 10^4^ MOI, 5 days after transduction. Images of 3T3-L1 cells taken with the IncuCyte S3. Figure S5. Confocal fluorescence images of the AAV transduced interscapular adipose tissue. Figure S6. (a) Undifferentiated, (b) differentiated and (c) Nile Red stained 3T3-L1 cells.
