# Supplementary material for: Assessment of Adipocyte Transduction Using Different AAV Capsid Variants
Source: Pharmaceuticals (Basel). 2024 Sep 18;17(9):1227. doi: 10.3390/ph17091227 (PMC11435061; doi:10.3390/ph17091227)
Supplement: Supplementary file 1 [file pharmaceuticals-17-01227-s001.zip › Figure S3.pdf]

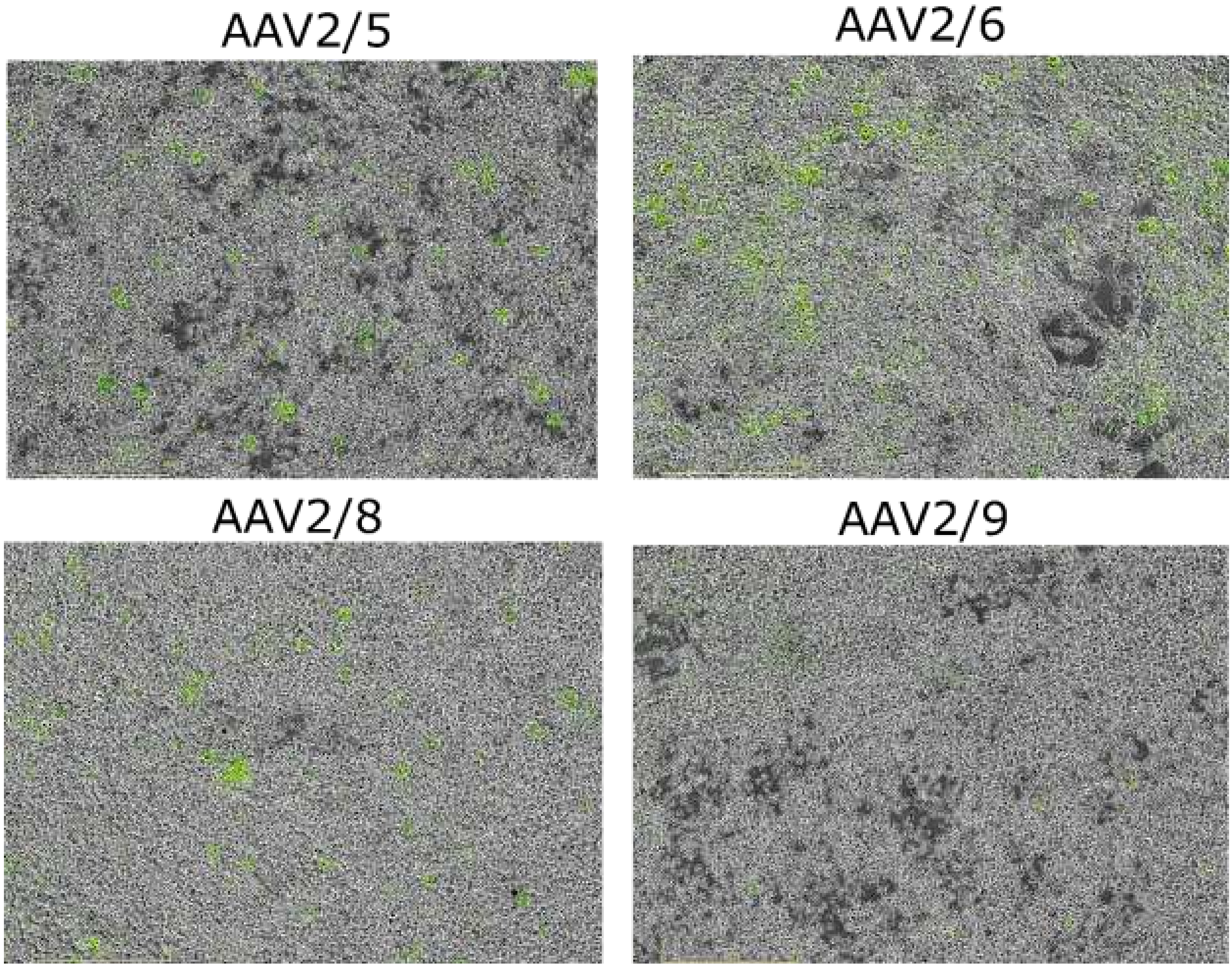

Figure S3. 3T3-L1 cells transduced cells with different AAV serotypes with further induction of differentiation. AAV concentration – $16 \cdot 10^4$ MOI, 10 days after transduction. Images of 3T3-L1 cells taken with the IncuCyte S3.
