# Supplementary material for: Assessment of Adipocyte Transduction Using Different AAV Capsid Variants
Source: Pharmaceuticals (Basel). 2024 Sep 18;17(9):1227. doi: 10.3390/ph17091227 (PMC11435061; doi:10.3390/ph17091227)
Supplement: Supplementary file 1 [file pharmaceuticals-17-01227-s001.zip › Figure S4.pdf]

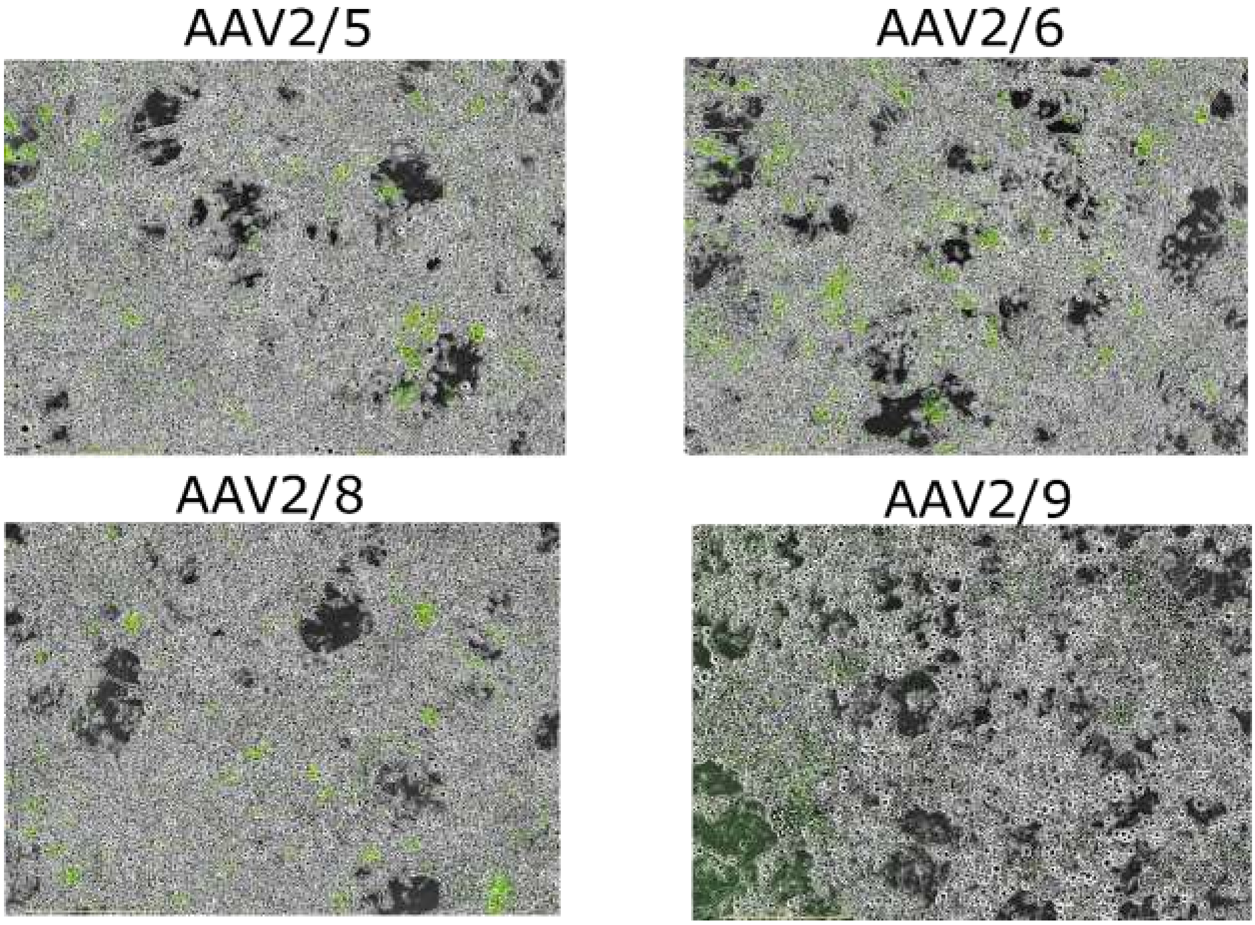

Figure S4. Differentiated 3T3-L1 cells transduced cells with different AAV serotypes. AAV concentration – $4 \cdot 10^4$ MOI, 5 days after transduction. Images of 3T3-L1 cells taken with the IncuCyte S3.
