# Supplementary figures and images for: Assessment of Adipocyte Transduction Using Different AAV Capsid Variants
Source: Pharmaceuticals (Basel). 2024 Sep 18;17(9):1227. doi: 10.3390/ph17091227 (PMC11435061; doi:10.3390/ph17091227)

(a)

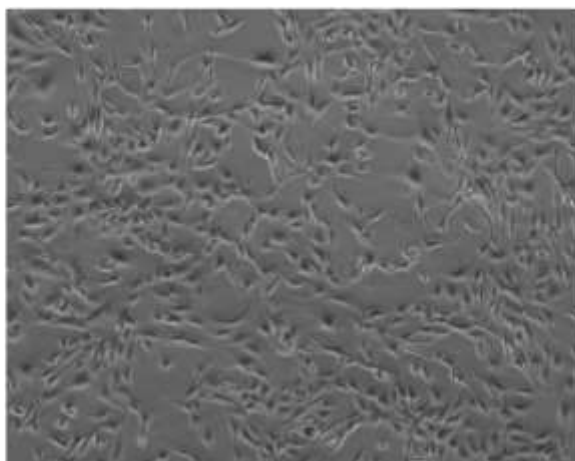

(b)

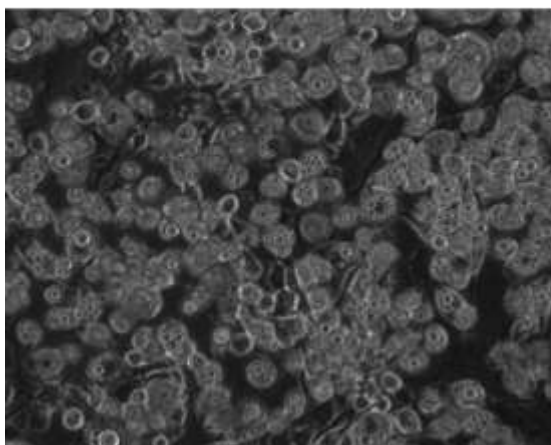

(c)

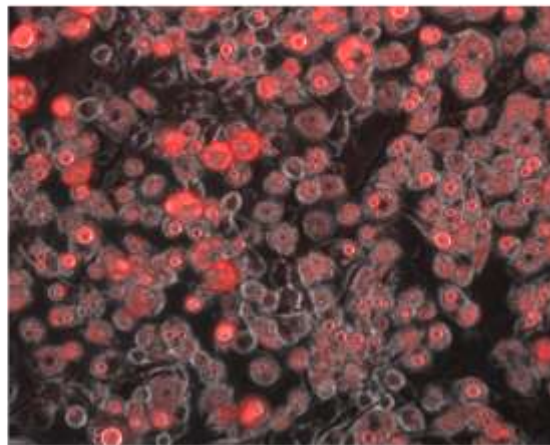

**Figure S6.** (a) Undifferentiated, (b) differentiated and (c) Nile Red stained 3T3-L1 cells

Supplement: Supplementary file 1 [file pharmaceuticals-17-01227-s001.zip › Figure S6.pdf]
